# Association of Insulin Resistance with Lipid Profile, Metabolic Syndrome, and Hormonal Aberrations in Overweight or Obese Women with Polycystic Ovary Syndrome

**Published:** 2015-03

**Authors:** Mehranghiz Ebrahimi-Mamaghani, Maryam Saghafi-Asl, Saeed Pirouzpanah, Akbar Aliasgharzadeh, Soudabeh Aliashrafi, Niloufar Rezayi, Mahzad Mehrzad-Sadaghiani

**Affiliations:** ^1^Nutrition Research Center, School of Nutrition, Tabriz, Iran; ^2^Department of Biochemistry and Diet Therapy, School of Nutrition, Tabriz, Iran; ^3^Department of Endocrinolgy, Imamreza Hospital, Tabriz, Iran; ^4^Student Research Committee, School of Nutrition, Tabriz University of Medical Sciences, Tabriz, Iran; ^5^Department of Gynecology and Obstetrics, Alzahra Hospital, Tabriz, Iran

**Keywords:** Abnormalities, Endocrinometabolic parameters, Insulin resistance, Lipid profile, Obesity, Polycystic ovary syndrome, Iran

## Abstract

This cross-sectional study was aimed to better clarify the associations of insulin resistance (IR) with endocrinometabolic parameters in polycystic ovary syndrome (PCOS). Anthropometric measurements, endocrine and metabolic profiles, and the presence of IR and metabolic syndrome (MetS) were assessed in 63 overweight or obese PCOS patients subdivided into insulin-resistant (IR) and insulin-sensitive (IS) groups. Fasting insulin concentration and HOMA-IR were higher (p<0.001), and quantitative insulin check index (QUICKI), glucose-to-insulin ratio (p<0.001), and high-density lipoprotein cholesterol (HDL-C) (p=0.012) were lower in IR group. MetS (p=0.034) and obesity (p=0.038) were more prevalent in IR group. For all PCOS patients, significant correlations of total cholesterol (TC) with dehydroepiandrosterone sulphate (DHEAS) (r=-0.27, p=0.031), HDL-C with QUICKI (r=0.26, p=0.036) were found. Partial correlations also showed significant associations between TG and BS2h (r=0.30, p=0.026) as well as TC and LH/FSH ratio (r=0.30, p=0.032). When the patients were divided into IR and IS groups, significant correlations of low-density lipoprotein cholesterol (LDL-C) with luteinizing hormone (LH) (r=0.50, p=0.017) as well as TC (r=0.42, p=0.043) and LDL-C (r=0.50, p=0.016) with LH/FSH ratio were observed in IR group. However, partial correlation suggested significant associations of HDL-C with testosterone (r=-0.35, p=0.049) as well as serum LDL-C (r=0.38, p=0.033), HDL-C (r=-0.32, p=0.047), and TC (r=0.34, p=0.056) with progesterone level only in the IS group. The findings of this study indicated that lipid abnormalities may occur in PCOS, irrespective of IR.

## INTRODUCTION

Polycystic ovary syndrome (PCOS) is often characterized by the manifestation of oligo/anovulation, clinical or biochemical hyperandrogenism and/or polycystic ovaries. PCOS affects 5% to 10% women of reproductive age (1). It is addressed that PCOS is a heterogeneous gynaecological syndrome associated with a wide range of endocrine and metabolic abnormalities, including hyperinsulinaemia, hyperglycaemia, glucose intolerance, dyslipidaemia, and obesity, which are regarded as the hallmark components of metabolic syndrome (MetS) (2).

Insulin resistance (IR) is considered the common cause of other aberrations in this disorder which affects the long-term health of PCOS patients (3). For instance, IR is considered to play a role in defected lipid profile. It is estimated that 70% of women with PCOS have at least one abnormal lipid constituent (4). Obese women with PCOS are more prone to dyslipidaemia, particularly elevated triglycerides (TG) and decreased high-density lipoprotein cholesterol (HDL-C) (5,6). Some studies are suggestive of significantly lower levels of HDL-C in PCOS women compared to weight-matched controls (7). However, in other investigations, no difference was observed in lipid profile between PCOS women and control participants (8,9).

It is estimated that approximately one-third of PCOS women also have MetS (10). In fact, PCOS is considered one of the ovarian manifestations of MetS (11). A study (10) showed that metabolic syndrome and its components are common in PCOS, especially among women with the highest BMIs and insulin levels. While obesity is regarded one of the putative factors leading to MetS, IR seems to contribute mainly to the link between PCOS and MetS (12). In addition, accumulating evidence indicates that women with MetS also exhibit hyperandrogenism (13), a well-established contributor to PCOS aetiology (14). Androgen in excess appears to affect independently, which further exacerbates the cardiometabolic aberrations in PCOS women (15). However, another study (6) showed that no correlation exist between lipid profile and gonadotrophic hormones or testosterone among PCOS patients. On the other hand, it was also suggested that metabolic disturbances were seemingly more pertinent to adiposity/insulin metabolism than to circulating androgen levels (16).

IR might also negatively correlate with dehydroepiandrosterone sulphate (DHEAS) concentration in PCOS patients (17). It is reported that obese women with PCOS have lower DHEAS levels compared to non-obese PCOS patients (18). Several studies have shown an inverse association between serum DHEAS and cholesterol levels (19,20), resulting in the high incidence of ischaemic heart disease (21,22).

The present study primarily focuses on the associations of IR with endocrinometabolic parameters among Iranian PCOS women. So far, most of the studies have discussed these interactions among PCOS patients with various body mass indices (BMIs) (6,23,24) whereas the present article discusses just on overweight or obese PCOS patients. The potential interrelationships of IR, obesity, endocrine disturbances, and MetS have been rarely studied altogether in the setting of PCOS. Therefore, this report was aimed to better clarify the mutual effects of these pathogenic abnormalities in overweight or obese PCOS patients subdivided into insulin-resistant (IR) and insulin-sensitive (IS) groups.

## MATERIALS AND METHODS

### Patient population

This cross-sectional study was conducted from January 2011 to August 2012 in Gynecology and Endocrinology Outpatient Clinics of Tabriz University of Medical Sciences. Sixty-three overweight or obese patients diagnosed with PCOS were recruited in this study. The research protocol was approved by the Ethics Committee of Tabriz University of Medical Sciences (ethical code=906). Written informed consent was obtained from all participants.

The diagnosis of PCOS was confirmed according to the revised Rotterdam criteria (25), in which the presence of any two out of the three following criteria was required: (i) oligo- and/or anovulation (<8 menstrual periods per year) (26); (ii) clinical and/or biochemical signs of hyperandrogenism, including hirsutism (Ferriman-Gallwey score >8); and (iii) polycystic ovaries on sonography (i.e. at least 1 ovary containing 12 or more peripheral follicles measuring 2-9 mm in diameter and/or ovarian volume of at least 10 mL) (27) and exclusion of other aetiologies (congenital adrenal hyperplasia, androgen-secreting tumours, and Cushing's syndrome). Vaginal or transabdominal pelvic sonography, as appropriate, was performed only on patients who did not fulfill the diagnostic criteria. The inclusion criteria were any PCOS patient diagnosed by the abovementioned criteria with age range between 17 and 37 years, BMI range between 25 and 39 kg/m^2^, and taking no medicines at least 2 months preceding the study. Any patients with a disease affecting metabolic parameters, including Cushing's syndrome, hypoglycaemia, diabetes mellitus, androgen-secreting tumours, or congenital adrenal hyperplasia, hyperprolactinaemia, hyperparathyroidism, thyroid disorders, and hypertension, were excluded from the study. Patients taking drugs that affect glucose or insulin metabolism, such as glucose-controlling drugs, contraceptives, glucocorticoids, beta-blockers, anti-coagulants, non-steroidal anti-inflammatory drugs (NSAIDS), anti-obesity drugs, multivitamins, and any dietary supplements within 2 months before the entry to the study were also excluded. In addition, history of being on a special diet, such as weight-losing diet during the 6 months before the onset of the study, was regarded an exclusion criterion.

The obtained data for medical history included age, intake of drugs, smoking and alcohol consumption, levels of physical activity. Dietary intake was measured using a 3-day food recall. Blood pressure (BP) was measured after a 10-min rest period, using digital automatic blood pressure monitor (Omron, Japan). Systolic BP above 130 mmHg and diastolic BP above 80 mmHg were regarded as hypertension (28). Subjects were weighed in light clothing without shoes. Height was measured to the nearest 0.1 cm, using a wall-mounted stadiometer. BMI was calculated as weight (kg) divided by the square of height (m). For further analysis, the patients were also subdivided into 2 groups of BMI: the overweight group (BMI 25-29.9 kg/m^2^) or the obese group (BMI ≥30 kg/m^2^) (29). Lipid profile and other parameters were also compared between these two subgroups. Waist-circumference (cm) was measured at a level midway between the lower rib margin and iliac crest (30).

### Laboratory measurements

After a 12-hour overnight fasting, 10 mL blood was obtained in the follicular phase of the menstrual cycle (i.e. serum progesterone level lower than 2.5 ng/mL) (31). In terms of high progesterone level, the whole measurements were repeated after one or two week(s). The whole blood samples were centrifuged at 3,000 rpm for 5 minutes. The samples were analyzed either immediately or during the first week after conservation at −20 °C.

Serum glucose, total cholesterol (TC), TG, and HDL were analyzed using the standard enzymatic method (Pars Azmoon kit, Pars Azmoon Inc., Tehran, Iran) (glucose: CV inter-assay=0.90%, TC: CV inter-assay=1.1%, TG: CV inter-assay=1.6%, and HDL: CV inter-assay=1.8%). LDL-C was calculated with the Friedewald (1972) Formula: LDL=[TC]-[HDL]-[TG]/5.0 (mg/dL). Levels of free testosterone (DiaMetra, Italy, CV inter-assay ≤10%), DHEAS (DRG Instruments GmbH, Germany, CV inter-assay=4.8%), and 17-OHP (DRG Instruments GmbH, Germany, CV inter-assay=6.7%) were measured using enzyme-linked immunosorbent assays (ELISAs) method. Total testosterone (CV inter-assay=5.3%), LH (CV inter-assay=3.8%), FSH (CV inter-assay=3.8%), prolactin (CV inter-assay=6.4%), progesterone (CV inter-assay=9.6%), and plasma insulin levels (CV inter-assay=3.9%) were all measured using chemiluminescence methods (Liaison®; DiaSorin S.P.A., Saluggia, Vercelli, Italy).

Hyperandrogenaemia was considered as either serum testosterone level above 2.08 nmol/L and/or serum DHEAS level above 7,800 nmol/L (32). Increased serum 17-OHP was defined in levels above 4.8 nmol/L to exclude congenital adrenal hyperplasia (33).

### Oral glucose tolerance test (OGTT) and evaluation of IR and MetS

The standard oral glucose tolerance test (OGTT) was performed two hours after administration of 75 g glucose for all the patients (34). Quantitative insulin check index (QUICKI), a simple marker for insulin sensitivity, was calculated as 1/(log fasting insulin × log fasting glucose in mg/dL) (35). The homeostasis model of insulin resistance (HOMA-IR) was calculated as [fasting plasma glucose concentration (mmol/L) × fasting serum insulin concentration (µU/mL)/22.5] (36). IR was defined as HOMA-IR value of ≥3.8 (37). Impaired glucose tolerance (IGT) was defined as an elevated fasting glucose (110 mg/dL ≤G_0_ ≤125 mg/dL) or an elevated 2-hour glucose (140 mg/dL ≤ G_120_ ≤199 mg/dL) (37). The patients were divided into insulin-resistant (IR) and insulin-sensitive (IS) groups. First, lipid profile and hormonal parameters were compared between the two groups. Then, the correlation of lipid profile with several metabolic parameters and hormonal profile were analyzed.

MetS was defined according to the National Cholesterol Education Program (NCEP) guidelines (38). Having at least three of the following criteria, individuals were diagnosed as MetS: increased waist-circumference (>88 cm), low serum HDL-C (<50 mg/dL in women), hypertriglyceridaemia (>150 mg/dL), hypertension (BP >130/80 mmHg), and high fasting blood glucose (>110 mg/dL).

### Statistical analysis

The Kolmogorov-Smirnov test was used in checking for the normality of data; all data were normally distributed. Data were expressed as mean±SD for continuous variables and as frequency (percentage) for categorical variables. Independent-sample *t*-test was used for comparing continuous variables, and chi-square or Fisher's exact test was used for categorical variables. Correlations between lipid profile and metabolic and hormonal parameters were examined using Pearson's correlation coefficients. Partial correlations were run to determine these associations after controlling for BMI and age. A p value of <0.05 indicated significance.

## RESULTS

None of the PCOS patients was taking drugs at least 2 months preceding the study. The mean age and BMI of PCOS patients were 26.9±5.7 years and 31.4±3.8 kg/m^2^ respectively. Thirty-six percent of overweight or obese PCOS patients had HOMA-IR ≥3.8 and constituted insulin-resistant group. Moreover, only 5 out of 63 PCOS patients were intolerant to glucose (142-215 mg/dL); however, none was diabetic. General characteristics and hormonal features of the patients in the IR and the IS groups are shown in Table 1.

More than half of the patients were similarly hyperandrogenic in the IR and IS groups (p=0.550). Around 80% of the IR and IS groups had hirsutism. The two groups had similar oligo/anovulation pattern (95% in both groups).

**Figure 1.**
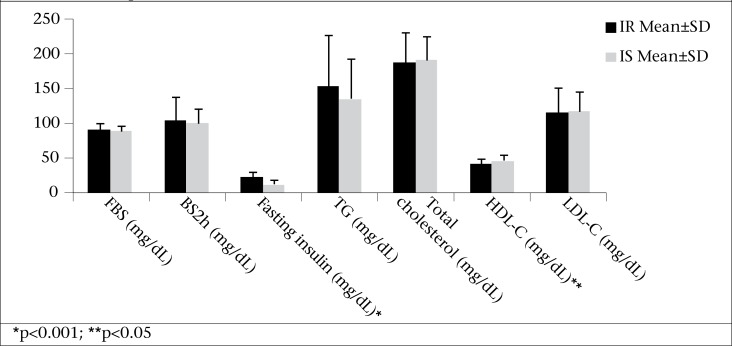
Mean±SD metabolic parameters between insulin-resistant (IR) and insulin-sensitive (IS) PCOS patients

**Figure 2.**
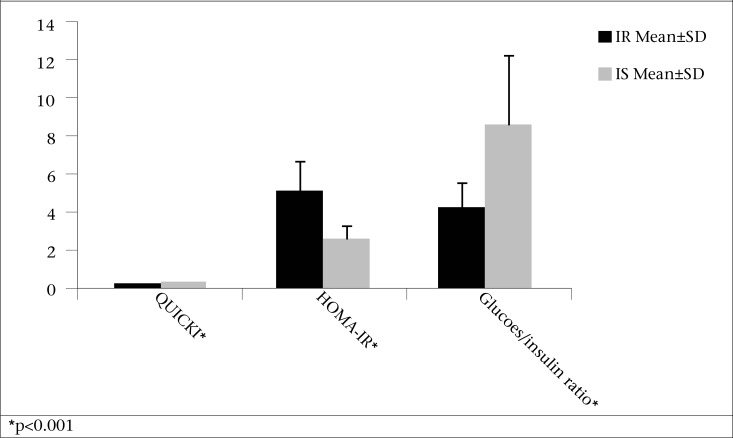
Mean±SD of QUICKI, HOMA-IR, and glucose-to-insulin ratio between insulin-resistant (IR) and insulin-sensitive (IS) overweight or obese PCOS patients

The IR and IS groups did not differ in terms of age and systolic or diastolic blood pressure (Table 1). Obesity was significantly more prevalent in the IR compared to the IS group (77% vs 50%, p=0.038) (data not shown). However, the mean BMI was similar between the two groups (32.27±3.46 in the IR vs 30.91±3.88 kg/m^2^ in the IS group, Table 1, p=0.175). Waist-circumference was non-significantly higher in the IR group compared to the IS group (p=0.072). There was no significant difference in energy intake, using a 3-day food recall, between the two groups (2,382 calories/day in the IR vs 2,355 calories/day in the IS group). Fasting insulin concentration (Figure 1) and HOMA-IR (Figure 2) were significantly higher (p<0.001), and QUICKI and glucose-to-insulin ratio were significantly lower (p<0.001) in the IR compared to the IS group (Figure 2). HDL-C was also significantly lower in the IR vs IS group (41.78±7.54 in the IR vs 47.25±8.38 in the IS group) (p=0.012) (Figure 1).

For further analysis, a comparison of lipid profile, metabolic and hormonal parameters was made between the two subgroups of overweight and obese PCOS patients. A significant difference was observed in fasting insulin concentration (18.42±9.61 mIU/mL in the obese vs 13.43±4.42 mIU/mL in the overweight group, p=0.008). HOMA-IR was significantly different between the two subgroups (3.86±1.92 in the obese vs 3.04±1.07 in the overweight group, p=0.036). HOMA-IR >3.8 (p=0.038) and one of the MetS components, i.e. waist-circumference (p<0.001), were significantly more prevalent among the obese PCOS patients. Other parameters were more or less similar between overweight and obese PCOS patients (data not shown).

The frequency of MetS and all of its individual components were higher in the IR compared to the IS group. Nearly half of the IR patients compared to 20% of the IS patients had MetS. However, only the frequency of MetS (p=0.034) differed significantly between the IR and the IS groups (Table 2).

**Table 1.** General characteristics and hormonal features between IR and IS group of PCOS patients

Variable	IR group (N=23) (Mean±SD)	IS group (N=40) (Mean±SD)	p value*
Age range (17-37 years)	26.96±6.58	26.80±5.29	0.918
Blood pressure (mmHg)			
Systolic	113.17±7.73	111.12±9.34	0.443
Diastolic	76.35±9.70	72.50±9.43	0.184
BMI (kg/m^2^)	32.27±3.46	30.91±3.88	0.175
WC (cm)	96.20±7.67	93.19±8.88	0.205
Calorie intake (kcal)	2,382.17±530	2,355.91±655	0.630
Fat intake (g)	91.60±43.18	79.87±41.11	0.317
FSH (U/L)	5.72±1.97	6.40±1.60	0.143
LH (U/L)	7.99±3.90	9.52±4.39	0.172
LH/FSH ratio	1.59±1.03	1.56±0.80	0.874
Testosterone (ng/mL)	0.64±0.17	0.68±0.25	0.467
Free testosterone (ng/mL)	1.40±0.93	1.31±0.97	0.740
DHEAS (µg/dL)	135.45±57.65	126.10±74.48	0.612
Progesterone (ng/mL)	1.56±0.65	1.38±0.62	0.297
17-OHP (nmol/L)	1.09±0.51	0.90±0.68	0.476

*t-test

IR=Insulin-resistant

IS=Insulin-sensitive

BMI=Body mass index

WC=Waist-circumference

FSH=Follicle-stimulating hormone

LH=Luteinizing hormone

DHEAS=Dehydroepiandrosterone sulphate

17-OHP=17-hydroxy-progesterone

Neither the IR nor the IS patients had significant differences in hormonal parameters (Table 1). For all PCOS patients, DHEAS concentration correlated inversely with TC level (*r*=-0.27, p=0.031); it became non-significant after adjustment for BMI and age. Among IR markers, only QUICKI significantly correlated with HDL-C level (*r*=0.26, p=0.036), which was non-significant after adjustment (*r*=0.24, p=0.072). Further analysis with partial correlations showed a significant association between TG and BS2h (*r*=0.30, p=0.026) as well as between TC and LH/FSH ratio (*r*=0.30, p=0.032). We also tried to find such correlations in subgroups of PCOS population (i.e. the IR and the IS groups) (Table 3-6). There was no correlation between serum insulin concentration and lipid profile in any of the groups. Other metabolic variables did not significantly correlate with lipid profile (Table 3), even after adjusting for the confounders (Table 5). However, regarding hormonal parameters, LH concentration correlated significantly with LDL-C (*r*=0.50, p=0.017) whereas non-significantly with total cholesterol (*r*=0.37, p=0.078) in the IR group (Table 4). This association became non-significant after adjustment (Table 6). The marginal correlation of FSH with HDL-C (*r*=0.41, p=0.051) in the IR group (Table 4) became significant (*r*=0.58, p=0.018) after controlling for the confounders (Table 6). Moreover, LH/FSH ratio correlated significantly with TC (*r*=0.42, p=0.043) and LDL-C (*r*=0.50, p=0.016) in the IR group (Table 4), which were non-significant after adjustment (Table 6). The reverse correlation of testosterone with HDL-C was also marginal (*r*=-0.30, p=0.059) in the IS group (Table 4), which became significant (*r*=-0.35, p=0.049) after controlling for the confounders (Table 6). Further analysis with partial correlation test indicated that there were significant associations of serum progesterone level with LDL-C (*r*=0.38, p=0.033), HDL-C (*r*=-0.32, p=0.047), and TC (*r*=0.34, p=0.056) only in the IS group (Table 6).

**Table 2.** Frequency of the metabolic syndrome components between IR and IS Group of PCOS patients

Variable	IR group (N=23) Number (%)	IS group (N=40) Number (%)	p value*
WC ≥88 cm	18 (85.7)	26 (66.7)	0.072
Hypertriglyceridaemia	10 (43.5)	15 (37.5)	0.188
Low HDL-C level	19 (82.6)	27 (67.5)	0.105
Hypertension	0 (0)	0 (0)	-
Fasting hyperglycaemia	3 (13)	5 (12.5)	0.301
Metabolic syndrome	10 (43.5)	8 (20)	0.034

*Chi-square test

IR=Insulin-resistant

IS=Insulin-sensitive

WC=Waist-circumference

HDL-C=High-density lipoprotein cholesterol

**Table 3.** Pearson's correlation coefficients for the associations of lipid profile with metabolic parameters in IR and IS group of PCOS patients

Variable	IR group (N=23)				IS group (N=40)			
	TG	TC	HDL-C	LDL-C	TG	TC	HDL-C	LDL-C
FBS	-0.22	-0.24	-0.02	-0.20	0.009	0.18	-0.03	0.22
BS2h	0.12	-0.29	-0.11	-0.37	0.13	-0.03	-0.12	-0.05
Fasting insulin	0.04	0.09	0.12	0.07	-0.01	-0.18	-0.13	-0.17
QUICKI	-0.008	-0.04	-0.09	-0.02	-0.22	0.12	0.10	0.20
Glucose/Insulin ratio	-0.17	-0.21	-0.05	-0.17	-0.15	0.22	0.10	0.29

IR=Insulin-resistant

IS=Insulin-sensitive

FBS=Fasting blood sugar

BS2h=Blood sugar 2 hours after 75 g oral glucose

QUICKI=Quantitative insulin check index

TG=Triglyceride

TC=Total cholesterol

HDL-C=High-density lipoprotein cholesterol

LDL-C=Low-density lipoprotein cholesterol

p values are non-significant for all the parameters

**Table 4.** Pearson's correlation coefficients for the associations of lipid profile with hormonal parameters in IR and IS group of PCOS patients

Variable	IR group (N=23)				IS group (N=40)			
	TG	TC	HDL-C	LDL-C	TG	TC	HDL-C	LDL-C
LH	-0.21	0.37	0.18	0.50*	0.13	0.03	-0.07	0.006
FSH	-0.38	-0.30	0.41**	-0.27	0.11	-0.16	-0.06	-0.21
LH/FSH ratio	0.14	0.42*	-0.22	0.50*	0.07	0.20	0.02	0.19
Testosterone	-0.13	-0.16	-0.07	-0.13	0.13	-0.001	-0.30**	0.03
Free testosterone	0.23	0.18	0.007	0.16	0.17	0.008	-0.03	-0.14
DHEAS	-0.24	-0.24	-0.07	-0.17	-0.04	-0.3	-0.23	-0.26
Progesterone	-0.11	0.07	0.22	0.08	0.12	-0.40	-0.28	-0.02

IR=Insulin-resistant

IS=Insulin-sensitive

LH=Luteinizing hormone

FSH=Follicle-stimulating hormone

DHEAS= Dehydroepiandrosterone sulphate

TG=Triglyceride

TC=Total cholesterol

HDL-C=High-density lipoprotein cholesterol

LDL-C=Low-density lipoprotein cholesterol

*p<0.05

**p=0.05 (marginally significant)

**Table 5.** Partial correlation coefficients of lipid profile with metabolic parameters in IR and IS group of PCOS patients (controlling for the effects of BMI and age)

Variable	IR group (N=23)				IS group (N=40)			
	TG	TC	HDL-C	LDL-C	TG	TC	HDL-C	LDL-C
FBS	0.003	0.006	-0.200	0.051	-0.086	0.101	-0.003	0.146
BS2h	0.450	0.263	-0.193	0.157	-0.03	-0.138	-0.090	-0.109
Fasting insulin	-0.004	-0.046	0.173	-0.009	0.215	-0.146	-0.130	-0.210
QUICKI	0.007	0.106	-0.107	0.151	-0.146	0.126	0.176	0.144
Glucose/Insulin ratio	0.015	0.130	-0.171	0.192	-0.127	0.181	0.180	0.195

IR=Insulin-resistant

IS=Insulin-sensitive

FBS=Fasting blood sugar

BS2h=Blood sugar 2 hours after 75 g oral glucose

QUICKI=Quantitative insulin check index

TG=Triglyceride

TC=Total cholesterol

HDL-C=High-density lipoprotein cholesterol

LDL-C=Low-density lipoprotein cholesterol

p values are non-significant for all the parameters

**Table 6.** Partial correlation coefficients of lipid profile with hormonal parameters in IR and IS group of PCOS patients (controlling for the effects of BMI and age)

Variable	IR group (N=23)				IS group (N=40)			
	TG	TC	HDL-C	LDL-C	TG	TC	HDL-C	LDL-C
LH	-0.376	0.195	0.248	0.362	0.267	0.204	-0.153	0.158
FSH	-0.380	-0.122	0.582*	-0.108	0.162	-0.143	0.036	-0.244
LH/FSH ratio	0.200	0.318	-0.362	0.385	0.20	0.346	-0.103	0.331
Testosterone	-0.072	-0.001	-0.153	0.262	0.125	0.119	-0.357*	0.196
Free testosterone	0.337	0.320	-0.102	0.070	0.131	0.010	0.051	-0.062
DHEAS	-0.145	-0.070	0.009	-0.019	-0.035	0.043	-0.141	0.112
Progesterone	-0.120	0.180	0.339	0.200	0.293	0.346**	-0.375*	0.384*

IR=Insulin-resistant

IS=Insulin-sensitive

LH=Luteinizing hormone

FSH=Follicle-stimulating hormone

DHEAS=Dehydroepiandrosterone sulphate

TG=Triglyceride

TC=Total cholesterol

HDL-C=High-density lipoprotein cholesterol

LDL-C=Low-density lipoprotein cholesterol

*p<0.05

**p=0.05 (marginally significant)

## DISCUSSION

The present study was aimed to investigate the associations of IR with endocrinometabolic parameters in overweight or obese PCOS patients. The results showed that fasting insulin concentration and HOMA-IR were higher (p<0.001); QUICKI and glucose-to-insulin ratio (p<0.001) and HDL-C (p=0.012) were lower in IR group. MetS (p=0.034) and obesity (p=0.038) were more prevalent in the IR group.

The prevalence of IR was 36.5% among the PCOS patients. This is higher than that (24.9%) reported previously in a large-scale study of PCOS patients conducted in Isfahan, Iran, with the same cutoff point for IR (39). According to our findings, IR is, at least to some degree, pertinent to obesity and not attributed solely to PCOS. In our study, insulin concentration and HOMA-IR were significantly higher in the obese PCOS patients compared to the overweight women (data not shown). On the other hand, the mean BMI and the prevalence of obesity (p=0.038) were higher in the IR compared to the IS patients, a finding which is consistent with previous studies (6,23,24). In fact, abdominal obesity can result in higher insulin concentration (40); and the resultant hyperinsulinaemia may encourage further obesity (41). Although IR occurs in obese as well as lean subjects with PCOS (42), any degree of obesity is liable to trigger reduced insulin sensitivity.

The IR and IS groups differed significantly concerning HOMA-IR, QUICKI, and glucose-to-insulin ratio. Lipid abnormalities were more common in the IR than the IS group. Among lipid profile, only the level of HDL-C was lower in the IR group, which is in line with prior studies (9,23,24,43). In the study conducted by Robinson *et al.* (9), the authors suggested that low HDL-C was associated with insulin sensitivity rather than BMI. In fact, IR seems to contribute to dyslipidaemia partly through lipolysis stimulation and altered expression of lipoprotein lipase and hepatic lipase (44). In our study, elevated LDL-C level was not found in the insulin-resistant patients as in the study by Kalra *et al.* (23) who compared lipid profile between insulin-resistant and non insulin-resistant PCOS groups. In another study, no significant correlation was observed between HOMA-IR and LDL-C (43).

In our research, hypertriglyceridaemia and hypercholesterolaemia—though higher in the IR patients—were not significantly different between the two groups. This finding contradicts with the results of Kalra *et al.* (23) in which the cutoff point of 4.5 was used for the diagnosis of insulin resistance. Moreover, the IS group was of normal BMI and the IR group were overweight whereas both of our groups were basically overweight or obese PCOS patients. In a study by Holte *et al.* (8), in which obese and non-obese PCOS patients were compared, the authors found that plasma free fatty acid concentrations dramatically increased in obese women with PCOS. This was closely associated with the lower insulin sensitivity and lower glucose tolerance in these women. Despite these broad metabolic abnormalities, the lipoprotein lipid profile was not significantly more abnormal in obese women with PCOS than in their weight-matched controls.

The prevalence of MetS in the IR vs IS PCOS patients was significantly higher (43.5% vs 20%) (p=0.034), which is consistent with preceding reports of one-third to one-half of the affected women (24,45). All the individual components of MetS were more common in the IR compared to the IS group as in the study by El-Mazny *et al.* (24), except for hypertension. As we recruited no hypertensive patient in the study, this item was not observed among the patients. Overall, these metabolic aberrations compromise the health of PCOS women by putting them at higher risk of cardiometabolic diseases (24).

Among IR markers, only QUICKI significantly correlated with HDL-C level (*r*=0.26, p=0.036) in all PCOS patients; however, it was non-significant after adjusting for the confounders. Further analysis among the IR and IS patients revealed significant correlations between lipid profile and obesity markers only in the IS group (data not presented). This finding indicates that the presence of IR may not necessarily lead to lipid abnormalities in PCOS as found in a prior study (6).

In the present research, the mean levels of basal serum LH and FSH were found to be lower in the IR compared to the IS patients; however, the difference was not significant. This could be due to the comparison of hormonal profile between the two groups with similar BMI. By contrast, in the study of Mor *et al.* (46), in which the level of LH was significantly lower in the IR group, patients with IR were significantly more obese than IS group (p<0.05). Obesity in PCOS has been shown to be associated with an attenuation of the LH pulse amplitude (47). In the present report, the ratio of LH/FSH did not differ between the IR and IS group. However, LH/FSH ratio correlated significantly with TC (*r*=0.30, p=0.032) in all PCOS patients after controlling for the confounders. Supporting this finding, a study (48) among nationally-representative sample of post-menopausal women showed that markers of dyslipidaemia, which are characteristics of PCOS-related morbidities, were also significantly associated with LH/FSH ratio. Similarly, in the IR group, LH/FSH ratio was a significant correlate of TC (*r*=0.42, p=0.043) and LDL-C (*r*=0.50, p=0.016); however, their association became non-significant after adjustment. This finding merits further investigation.

Lower testosterone level observed among the IR patients in this study may result from the low levels of LH in those patients as reported by Mor *et al.* (46). The observation of low LH in obese insulin-resistant women is in line with the results of previous studies (49,50), offering two distinct phenotypes for PCOS—a low-LH and high-insulin group and a high-LH and low-insulin group (51), further necessitating the designation of PCOS patients into IR and IS groups. Such division is not currently considered among the criteria which define PCOS.

The results from partial correlation suggested an inverse correlation between testosterone and HDL-C (*r*=-0.35, p=0.049) in the IS group as well as between FSH and HDL-C (*r*=-0.58, p=0.018) in the IR group. These findings are in contrast with the study of Meirow and co-workers (6) in which no correlations were found between lipid profile and any of the gonadotrophic hormones or testosterone among PCOS patients. In hirsute women, total and free testosterone levels correlated with triglycerides (*r*=0.72, p<0.05; *r*= 0.55, p<0.01 respectively) and HDL-C (*r*=-0.55; p<0.05; *r*=0.68, p<0.05 respectively) (52). In another study (53) of 430 healthy women, FSH had positive correlations with TC (*r*=0.13) and HDL-C (*r*=0.13) in post-menopausal women. Testosterone had no correlation with lipid profile. However, exogenous testosterone is reported to affect HDL-C negatively via hepatic lipase (HL), an enzyme which enhances the clearance of HDL-C (54).

Concerning adrenal steroidogenesis, there was an inverse significant relationship between DHEAS and cholesterol level (*r*=-0.27, p=0.031) in all PCOS patients as in prior studies (6,19,20) but this association was no longer significant after adjustment for BMI and age. DHEAS may directly affect insulin sensitivity by increasing insulin-binding to its own receptor (55). Evidence suggests that DHEA may also be associated with IR and hyperinsulinaemia through its relation with obesity (56), which was shown in the present study. Further analysis with partial correlation indicated that serum progesterone level had direct correlations with LDL-C (*r*=0.38, p=0.033) and TC (*r*=0.34, p=0.056), and inverse correlation with HDL-C (*r*=-0.32, p=0.047) only in the IS group. This finding shows that the level of serum progesterone can affect lipid profiles in PCOS patients of the IS group. It is also in accordance with another study which was suggestive of the favourable effect of progesterone on HDL-C and LDL-C (57).

A relatively limited sample was included in the present study due to taking into account a vast number of effective confounders as inclusion criteria. The controversies which exist in different studies may result from different diagnostic criteria used for PCOS, inclusion of women with different BMIs, and probable use of progestin for menstrual induction preceding the study. However, in the present investigation, diagnosis of PCOS was based on the Rotterdom criteria, widely used in most researches. Moreover, only overweight or obese patients at a limited age range (i.e. 17-37 years) were recruited. Finally, no hormonal treatment (i.e. progestin) was used for menstrual induction before the study.

### Conclusions

Regarding the higher prevalence of obesity and MetS in the PCOS patients of the IR group, screening such women to prevent further cardiometabolic complications seems warranted. Furthermore, lipid abnormalities may occur in PCOS, irrespective of IR. To manage the disease, the complicated interrelationships among lipid profile, obesity, IR, MetS, and hormonal parameters should be considered.

## ACKNOWLEDGEMENTS

The data of this paper are extracted from a PhD thesis (No. 24/D). We acknowledge Research Vice-Chancellor as well as Student Research Committee of Tabriz University of Medical Sciences for their funding. We thank the staff of Gynecology and Endocrinology Clinics of Tabriz University of Medical Sciences for their cooperation. We also appreciate all patients for participation in the study.

**Conflict of interest:** Authors declare no conflicts of interest.
